# An Aqueous Extract of Marine Microalgae Exhibits Antimetastatic Activity through Preferential Killing of Suspended Cancer Cells and Anticolony Forming Activity

**DOI:** 10.1155/2016/9730654

**Published:** 2016-08-31

**Authors:** Syam Prakash Somasekharan, Amal El-Naggar, Poul H. Sorensen, Yuzhuo Wang, Hongwei Cheng

**Affiliations:** ^1^Centre for Biosciences and Biomedical Engineering, Indian Institute of Technology, Indore, India; ^2^Department of Molecular Oncology, British Columbia Cancer Research Centre, Vancouver, BC, Canada; ^3^Department of Urologic Sciences, University of British Columbia, Vancouver, BC, Canada; ^4^Department of Experimental Therapeutics, British Columbia Cancer Research Centre, Vancouver, BC, Canada

## Abstract

Research on marine natural products as potential anticancer agents is still limited. In the present study, an aqueous extract of a Canadian marine microalgal preparation was assessed for anticancer activities using various assays and cell lines of human cancers, including lung, prostate, stomach, breast, and pancreatic cancers, as well as an osteosarcoma.* In vitro*, the microalgal extract exhibited marked anticolony forming activity. In addition, it was more toxic, as indicated by increased apoptosis, to nonadherent cells (grown in suspension) than to adherent cells.* In vivo*, an antimetastatic effect of the extract was observed in NOD-SCID mice carrying subrenal capsule xenografts of PC3 prostate cancer cells. The results of the present study suggest that the antimetastatic effect of the aqueous microalgal extract is based on inhibition of colony forming ability of cancer cells and the preferential killing of suspended cancer cells. Further research aimed at identification of the molecular basis of the anticancer activities of the microalgal extract appears to be warranted.

## 1. Introduction

Cancer is one of the most life-threatening diseases worldwide. In 2012, about 14 million new cases of cancer occurred globally, resulting in about eight million deaths [1]. Although many modern designed anticancer drugs can extend the survival time of patients, the substantial side effects of such drugs usually severely compromise the quality of life of the patients. Plants have been a prime source of conventional, clinically useful drugs for the treatment of many forms of cancer and even if the actual plant-derived compounds did not serve as drugs, they provided leads for the development of potential novel agents [2]. As such, exploring plants for effective anticancer agents with relatively low side effects appears to provide an attractive strategy even in the modern age of anticancer drug development.

Marine plants have long served as a major and diverse source of numerous types of natural products [3–6]. Among the marine plants, marine algae (phytoplankton) constitute a substantial part of the ocean flora as the largest body for oxygen production and form the basis of the marine food chain [7]. Marine algae are found rich in many nutrients such as carbohydrates, amino acids, fatty acids, vitamins, pigments, and essential and trace minerals [8]. Consequently, they have long become valuable nutrition sources in the food and animal feeding industries; for example, several species of microalgae are commercially produced as supplements of unsaturated fatty acids [8] and single cell proteins [9]. In addition, marine algae are frequently used as food additives, flavouring and colouring agents [10–12]. Recently, many types of biologically active components have been identified in marine algae and used in medicinal applications. For example, sulfated polysaccharides (SPs) have been found enriched in the cell walls of marine brown algae [13, 14], exhibiting many potentially therapeutic activities including antiviral, antioxidant, antiallergic, anti-inflammatory, immunomodulatory, hypolipidaemic, hypoglycaemic, and anticoagulatory properties [15–21].

Anticancer activities of marine algae-derived products are also well documented. Examples include the growth inhibitory effects on leukemic cells of algal extracts of* Gracilaria corticata* and* Sargassum oligocystum* [22]; inhibition of proliferation of and induction of apoptosis in human oral cancer Ca9-22 cells by an ethanol extract of* Gracilaria tenuistipitata* [23]; sensitisation of human HT-29 colon carcinoma cells by* Plocamium telfairiae* extracts to caspase-dependent apoptosis [24]; and anticancer effects against colon cancer cells by extracts of brown* Laminaria japonica* and* Sargassum horneri* algae [22].

Here we report that an aqueous extract of a natural Canadian marine microalgal preparation has significant anticolony forming activity against multiple cancer cell lines. In addition, the extract preferentially induced apoptosis of suspended cells as distinct from adherent cells. Lastly, the extract showed antimetastatic activity in NOD-SCID mice bearing xenografts of PC3 prostate cancer cells.

## 2. Methods and Materials

### 2.1. Microalgal Material

Raw marine microalgal material (dried powder) was obtained from the Canada Marine Biotech Research Corp, BC, Canada; http://canadacellton.ca/.

### 2.2. Cell Culture

All human cell lines used in this study were purchased from the American Type Culture Collection (ATCC). A549, H460, PC3, DU145, and N87 cells were cultured in RPMI-1640 supplemented with 10% fetal bovine serum (FBS). MCF7, BxPC3, and MNNG cells were cultured in DMEM supplemented with 10% FBS. Cell lines were grown at 37°C in an atmosphere of 5% CO_2_/air. All media and FBS were purchased from GE Healthcare Hyclone, Logan, UT.

### 2.3. Microalgae Extract Preparation

To prepare an aqueous extract of marine microalgae, raw marine microalgal material (obtained from Canada Marine Biotech Research Corp, BC, Canada; http://canadacellton.ca/) was first suspended in distilled water at a concentration of 30 mg/mL. The microalgal suspension was then sonicated at 60% amplitude and 20 sec short bursts (×10) with 30 sec intervals for cooling. The sonicated suspension was then passed through a 25 gauge needle to release the cytosolic contents, followed by syringe filtration through 0.2 *μ*m filters. The filtrate was stored at 4°C as a stock solution.

### 2.4. Microscopy

To study the morphology of the microalgae, raw marine microalgae were gently suspended in PBS at a concentration of 1 mg/mL. A drop of the suspension was evenly spread on a slide and viewed under a phase contrast microscope at 40x objective; images were taken by camera. In analyzing tumor cell invasion and metastasis, primary tumor and lung tissues were processed and analyzed as previously described [25, 26]. Briefly, harvested tissues were formalin fixed and paraffin-embedded. The processed tissues were then subjected to serial sectioning and stained with haematoxylin-eosin (HE) and IHC with anti-human Ki67 antibody. The sections were then imaged under a microscope at 40x magnification (Axio Observer Z1; Carl Zeiss) and analyzed by AxioVision software.

### 2.5. Colony Forming Assay

For the assay, single cell suspensions of cultured cancer cells were prepared and cell numbers counted using a TC20 Automated Cell Counter (Bio-Rad, Hercules, CA). 1 × 10^3^ live cells were seeded in 6-well petri plates containing culture media, supplemented with microalgal extract at a range of concentrations. After 10 days of incubation, the media were removed from the plates and cells were washed with PBS. The clones were then fixed with glutaraldehyde (6.0% v/v), stained with crystal violet (0.5% w/v), washed with water, and dried at room temperature [27]. The colonies formed were counted using a stereomicroscope and imaged with a camera.

### 2.6. PolyHEMA Coating of Culture Plates and Apoptosis Assays

To keep the cells in a suspended, nonadherent state, culture plates were used which had been coated with polyHEMA [Poly(2-hydroxyethyl methacrylate), Sigma-Aldrich], using 4 mL of polyHEMA (10 mg/mL in ethanol) per plate [28]. Apoptosis assays were conducted as described previously [29]. 1 × 10^5^ cells were seeded in polyHEMA-coated or noncoated 6-well petri plates containing growth medium supplemented with microalgal extract at 0, 1, and 5 mg/mL concentrations. After 24 h of incubation, cells were collected, stained with annexin V-FITC and PI, and analyzed for apoptosis using FACS following a standard experimental protocol (BD Biosciences).

### 2.7. Cell Growth Inhibition Assays

A549, MCF-7, PC-3, DU-145, N87, BxPC3, H460, and MNNG cancer cells were seeded on 96-well plates. Cells (1 × 10^3^) of each line were incubated with microalgal extract at various concentrations (0, 1, 2, and 5 mg/mL) for 72 h. A standard MTT assay protocol [30] was used to determine the relative cell numbers and growth inhibitory effects of the microalgal extract.

### 2.8. *In Vivo* Experiment

An experiment involving xenograft-bearing mice was conducted with a protocol approved by the University of British Columbia Animal Care Committee. The detailed tumor implantation procedure used has previously been described [26]. In brief, each PC3 xenograft cell block was prepared by mixing 1 × 10^6^ PC3 cells with 40-*μ*L of 5 × DMEM and 10 *μ*L of rat-tail collagen gel. After incubation for 30 min at 37°C, solidified xenograft cell blocks were transferred to the animal facility in a sterile container for implantation. Sixteen 8-week-old NOD-SCID male mice were used for implantation of the premade cell blocks under the renal capsule membrane in the left-side kidney of each mouse. Three weeks after implantation, 4 mice received oral administration of aqueous microalgal extract (30 mg/mL) at a dosage of 300 mg/kg, from Monday to Friday for 3 weeks. Four control mice were treated with sterile water using the same schedule and mode of administration. At harvest, mice were euthanized using the Facility's standard SOP and all primary tumor tissues and lungs were collected for further analysis.

### 2.9. Immunohistochemistry, Proliferation Index, and Metastasis Quantification Assays

Anti-human Ki67 immunohistochemistry staining was performed using our lab's standard IHC protocol as previously described [25, 26]. For assessing the cell proliferation rate in primary tumors, Ki67-stained proliferating cells were documented by analyzing 10 randomly chosen high-power fields (40x) per tumor section (*n* = 6). The ImageJ plug-in “ImmunoRatio” was used to directly calculate the percentage of Ki67-positive cells in each image. For assessing pulmonary metastases, lung tissue sections from each host mouse were histologically examined for all Ki67-positive human-origin cancer cell foci under 20x magnification. Lung metastatic foci bigger than 50 microns were counted under 20x magnification across whole section areas. Lung surface areas were calculated using the counts of the total high-power fields (20x) in each section.

### 2.10. Statistical Analysis

Statistical analysis was performed using GraphPad Prism 6 (GraphPad Software, Inc., La Jolla, CA). The Student* t*-test was carried out to compare means between two groups. Two-way ANOVA followed by post hoc multiple comparison was applied to compare tumor proliferation rates and lung metastases. Results with a *p* value < 0.05 were considered statistically significant and are indicated by *∗* for *p* < 0.05, *∗∗* for *p* < 0.01, and ∗∗∗ for *p* < 0.001.

## 3. Results

### 3.1. Microscopic Examination of Marine Microalgae

**Figure 1:**
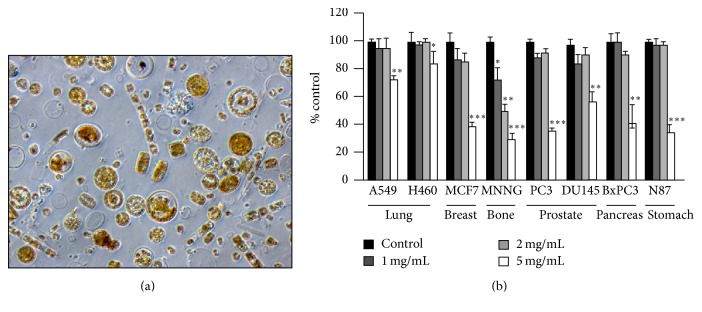
Microalgae extract suppresses the replication of adherent cancer cells. (a) Morphology of suspended marine microalgae (40x magnification). (b) Cells of multiple cancer cell lines were incubated with microalgal extract (0, 1, 2, and 5 mg/mL) for 72 h and the growth inhibition was determined using the MTT assay. The data represent results of 3 independent experiments. Mean values ± SD (error bars) are shown. ^*∗*^ *p* < 0.05; ^*∗∗*^ *p* < 0.01; ^*∗∗∗*^ *p* < 0.001.

A microalgal suspension was made from dried Canadian marine microalgae powder in PBS at a 1 mg/mL concentration and examined under a microscope (40x magnification) for morphology heterogeneity. The microalgal suspension was found to be highly heterogeneous, containing various types of microalgae (Figure 1(a)).

### 3.2. Aqueous Microalgal Extract Inhibits Cancer Cell Replication

Various cancer cell lines, that is, A549 and H460 (lung), MCF-7 (breast), MNNG (osteosarcoma), PC-3 and DU-145 (prostate), BxPC3 (pancreas), and N87 (gastric), were incubated with microalgal extract (at 0, 1, 2, and 5 mg/mL) for 72 h and cell proliferation was determined using the MTT assay. As shown in Figure 1(b), the microalgal extract did not significantly inhibit replication of the tested cell lines at 1-2 mg/mL except in the case of MNNG. At 5 mg/mL, the extract significantly inhibited the growth of all the cell lines.

### 3.3. Microalgal Extract Inhibits Colony Forming Ability of Cancer Cells

**Figure 2:**
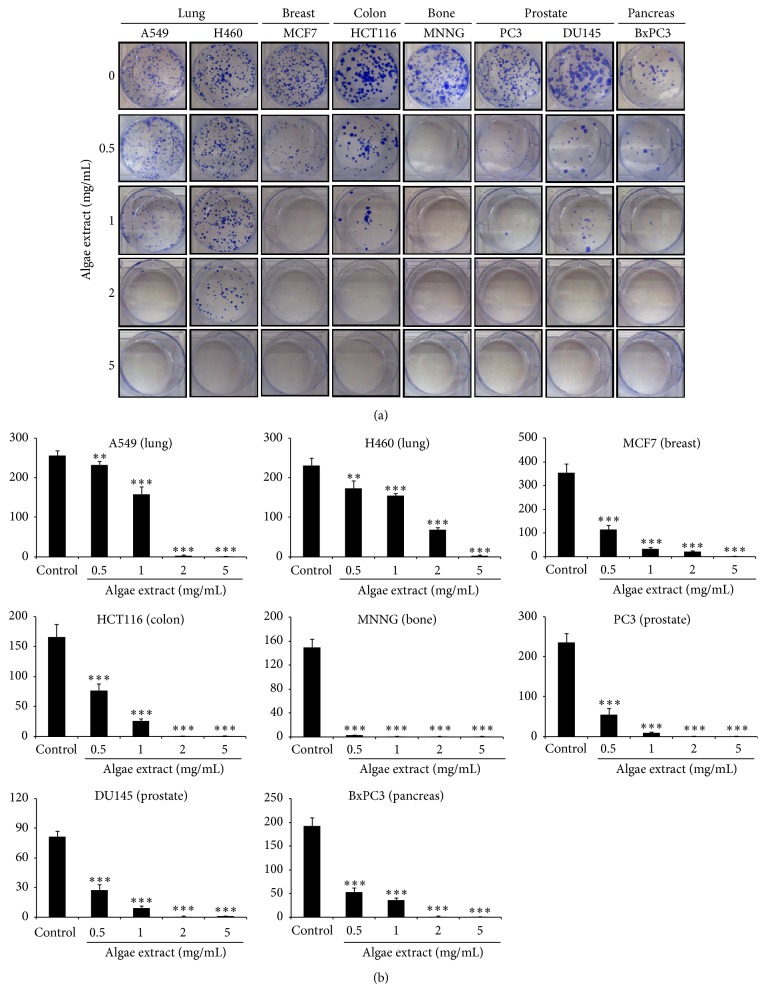
Colony forming assay of adherent cancer cells in response to treatment with microalgal extract. Cells of multiple cancer cell lines were incubated for 10 days with microalgal extract at the indicated concentrations. (a) Representative images show the clones formed under the various conditions. (b) The number of clones formed under each condition in (a) was counted and presented as histograms. The results are representative of three independent experiments. Mean values ± SD (error bars) are shown. ^*∗∗*^ *p* < 0.01; ^*∗∗∗*^ *p* < 0.001.

To examine the effect of the microalgal extract on colony forming ability of cancer cells we used the* in vitro* colony formation assay which is based on the ability of a single cell to grow into a colony of at least 50 cells [27]. Cells of the various cancer lines were seeded at 500 cells/mL in 6-well petri plates containing culture medium supplemented with microalgal extract at a range of concentrations [0 (vehicle) and 0.5–5 mg/mL]. After 10 days of incubation, the colonies formed under each condition were counted. As shown in Figures 2(a) and 2(b), the colony forming ability of all the tested cancer cell lines was significantly inhibited by the microalgal extract, even at the low concentration of 0.5 mg/mL, suggesting that the aqueous microalgal extract has a strong anticolony forming activity against multiple types of cancer cells.

### 3.4. Treatment with Microalgal Extract Reduces Metastasis in a Prostate Cancer Xenograft Model

**Figure 3:**
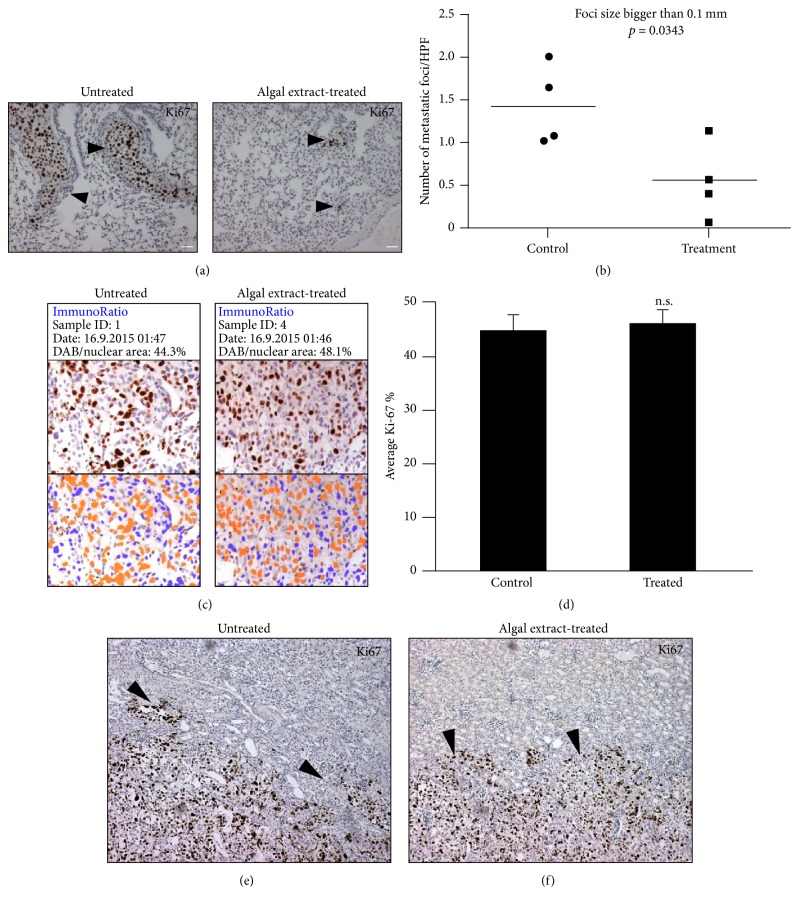
Microalgal extract inhibits metastasis of PC-3 prostate cancer cells. (a) Representative Ki67 IHC images showing metastatic foci in the lungs of treated and untreated mice carrying xenografts of PC-3 prostate cancer cells. (b) The graph shows the metastatic foci counts (>0.05 mm) in the lungs of both untreated and treated mice and their statistical comparison results. Bars, 100 *μ*m. (c) Primary tumors in control mice and microalgal extract-treated mice were stained with human Ki67 antibody as a cell proliferation biomarker. (d) The percentage of Ki67-positive PC-3 cells in each group was quantitated as a bar graph (right) by counting cells in 20x high-power fields of each tumor sample (*n* = 4 tumors per group). ((e) and (f)) Representative Ki67 IHC images show the degree of the primary tumor's local tissue invasiveness at the boundary between the primary tumor and the host kidney. Arrow heads show highly invasive growth patterns of the primary tumors in both the control and the microalgae extract-treated mice. Mean values ± SD (error bars) are shown. n.s.: not significant.

Of the various cell lines tested, the PC-3 prostate cancer cells showed a relatively high sensitivity to treatment with the microalgal extract (Figures 2(a) and 2(b)). Also, PC-3 cells have a relatively high metastatic potential [31]. In view of this we used PC-3 cells to examine whether the microalgal extract had an effect on the growth and metastasis of cancer cells* in vivo*. We choose NOD-SCID mice bearing subrenal capsule PC3 xenografts as a model, as it allows dynamic monitoring of tumor progression from various perspectives, such as primary tumor growth, tumor invasion of adjacent kidney tissues, and distant metastasis to the mouse host lungs in a time frame of only 4–6 weeks [25, 26]. Examination of the lungs of the extract-treated mice, relative to the controls, showed that the treatment with the microalgal extract had reduced the metastasis of the PC3 cells, as evidenced by the presence of significantly smaller lung metastatic foci (Figures 3(a) and 3(b)). In contrast, there was no difference between the control and treated mice in the cell proliferation rate of primary tumors as assessed with the Ki67 cell proliferation marker (Figures 3(c) and 3(d)). We also did not find a difference between the two groups in terms of local tissue invasion (Figures 3(e) and 3(f)). These results indicate that the decreased lung metastasis observed in the extract-treated mice was not related to the proliferation rate, nor to the tissue invasiveness of the cancer cells at the primary site. Also, during the experiment period, we did not observe any body weight loss and changes of vital signs in the treated mice, suggesting a nontoxic characteristic of the microalgae.

### 3.5. Microalgal Extract Preferentially Induces Apoptotic Cell Death of Suspended Cells

**Figure 4:**
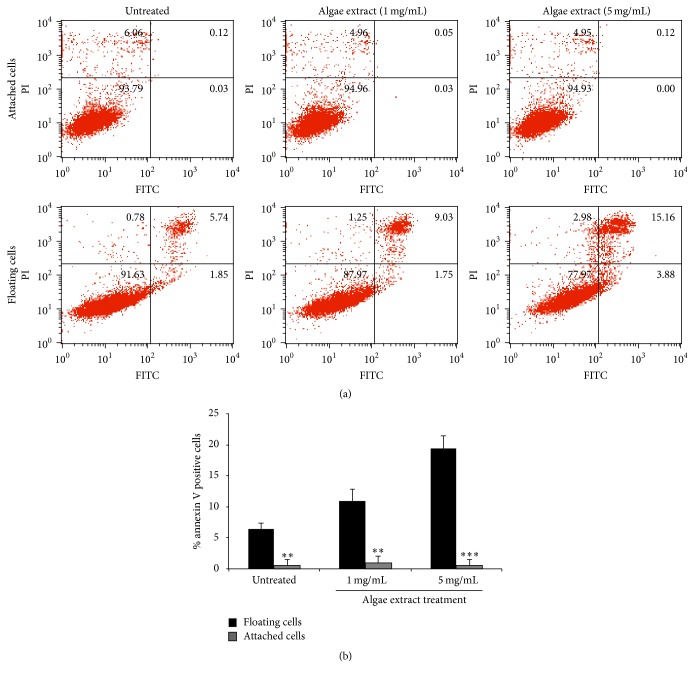
Microalgal extract preferentially kills suspended PC-3 cells. (a) FACS results showing the proapoptotic effect of the microalgal extract on adherent and suspended prostate cancer PC-3 cells. (b) The above data were quantified as bar graphs. Mean values ± SD (error bars) are presented. ^*∗∗*^ *p* < 0.01; ^*∗∗∗*^ *p* < 0.001.

Metastasis is reported to be usually positively related to enhanced proliferation and profound local tissue invasiveness of primary tumor cells [32, 33]. Although our* in vivo* results did not show a significant difference in these two parameters between primary PC-3 tumors of the microalgal-treated and the control mice (Figures 3(c), 3(d), and 3(e)), there was a significant reduction in lung metastasis in the extract-treated mice (Figures 3(a) and 3(b)). This interesting observation made us wonder if the reduced lung metastasis in the extract-treated mice was related to higher sensitivity of suspended PC-3 cancer cells to treatment with the extract. As anticipated (Figures 4(a) and 4(b)), treatment of suspended PC-3 cells with the microalgal extract at a low concentration (1 mg/mL) led to apoptosis, whereas adherent PC-3 cells did not undergo apoptosis when treated with a high concentration of the extract (5 mg/mL) for the same time period. Similar results were also obtained with other cell lines (date not shown). These findings demonstrate that the microalgal extract preferentially causes apoptotic cell death of suspended cancer cells.

## 4. Discussion

Cancer metastasis is responsible for over 90% of cancer patients' deaths and is the most challenging problem in cancer patient management [33]. Once cancer starts to metastasise, the disease becomes very difficult to control due to cancer cells widely spreading to other organs of the body along with evolving resistance to most anticancer therapies [32–34]. There is therefore a critical need for novel, metastasis-targeting drugs with relatively low side effects.

Although the idea of using natural medicinal products to prevent and treat cancer metastasis is widely welcomed, research in this area has been limited. Recently, use of marine natural products as potential anticancer agents has received more attention as increasing numbers of medicinal compounds of this type have been identified [22]. Among marine organisms, marine microalgae are considered to be of particular importance in view of their high availability and extensive biodiversity [8, 35]. Indeed, there is a growing body of scientific information indicating the therapeutic values of microalgal products in the management of chronic diseases, including cancer [17, 36–38]. Consequently, there is a compelling demand for exploring various microalgal products for anticancer activities and ultimately transferring them into new anticancer or cancer prevention methods.

In metastasis process, circulating tumor cells (CTCs) or circulating tumor microemboli (CTMs) develop via detachment from the primary tumor site and intravasation into the circulation system, by which they are carried to other organs of the body where some of them can form colonies and eventually metastatic lesions [33, 39, 40]. In the whole invasion-metastasis cascade, metastatic colonization is the final and is thought to be the dominant rate limiting step [41]. This is because, by concurrently solving microenvironmental incompatibilities and activating self-renewal pathways, only a small minority of disseminated CTC cells can succeed in completing the process of metastatic colonization and thereby generate macroscopic, clinically detectable metastases.

Our finding that treatment with an aqueous microalgae extract can reduce metastasis* in vivo* appears to be related to the preferential killing of suspended cancer cells and the anticolony forming properties of the microalgal extract. Based on these findings, the microalgal extract may be useful for future development of antimetastatic therapeutic agents with preferential CTCs/CTMs killing and inhibition of metastatic colonization activities. Therefore, further research aimed at identification of the molecular basis of the anticancer activities of the microalgal extract appears to be warranted.
